# Biodegradation of Doxylamine From Wastewater by a Green Microalga, *Scenedesmus obliquus*

**DOI:** 10.3389/fmicb.2020.584020

**Published:** 2020-11-03

**Authors:** Jiu-Qiang Xiong, Pengfei Cui, Shaoguo Ru

**Affiliations:** College of Marine Life Sciences, Ocean University of China, Qingdao, China

**Keywords:** pharmaceutical contaminants, microalgae, doxylamine, wastewater, bioremediation, biodegradation

## Abstract

Pharmaceutical contaminants (PCs) have been recognized as emerging contaminants causing unexpected consequences to environment and humans. There is an urgent need for development of efficient technologies to treat these PCs from water. The current study has investigated the removal capacity of a green microalgal species, *Scenedesmus obliquus*, for doxylamine, chemical oxygen demand (COD), and nutrients from real wastewater. Results have indicated that *S. obliquus* can grow well in the doxylamine-polluted wastewater with the achievement of 56, 78.5, 100, and 89% removal of doxylamine, COD, total nitrogen (TN), and total phosphorus (TP). Addition of 2 g L^−1^ bicarbonate enhanced the removal of doxylamine up to 63% and slightly inhibited the removal of COD. Decreased carbohydrate (28–26%) and increased protein content (30–33%) of the harvested biomass have been observed after cultivation in the wastewater. The current study has shown the feasibility of using microalgae-based biotechnologies for PC-contaminated wastewater.

## Introduction

Pharmaceuticals have been extensively used in human, and culturing of livestock, poultry, and fish to help and facilitate their lives. These high-biologically activated compounds cannot be efficiently degraded, and <70% of the uptaken drugs can be excreted into environment with urine and feces (Tran et al., 2018). Numerous countries including United States, China, Canada, Europeans, South Korea, and Japan have found their frequent occurrence in surface water, groundwater, wastewater, seawater, and soil with levels of ng L^−1^ to μg L^−1^ (Patel et al., 2019). These pharmaceutical contaminants (PCs) have been recognized as emerging contaminants since increasing evidences have demonstrated their unexpected consequences on ecological systems and health of humans. For example, PCs at environmental concentrations extended the lag phase of benthic microorganisms, inhibited bacterial denitrification, and reduced microbial diversity (Underwood et al., 2011). Potential permanent damage and consequent effects on coming generations can be induced by PCs demonstrated by their significant influence on uterus [Commission European (CE), 2016]. Existence of the PCs in environment also can generate drug-tolerant bacteria and antibiotic-resistant genes, which can be biomagnificated through food web and finally reach higher consumers (Martínez, 2008). There is an urgent need for effective treatment of such PCs from environments.

Traditional conventional technologies for wastewater treatment are highly effective for removal of chemical oxygen demand (COD); however, it shows limited performance to remove persistent PCs, total nitrogen (TN) and total phosphorus (TP; Matamoros et al., 2015). Different strategies such as advanced oxidation processes (AOPs), adsorption, bacterial degradation, and electrolysis have shown highly efficient performance of PC treatment (Patel et al., 2019). There are unexpected disadvantages with use of these advanced approaches. For example, more hazardous intermediates can be formed during AOPs and electrolysis (Yang et al., 2019); organic matrixes of the wastewater can significantly decrease the adsorption capacity (Ersan et al., 2017); and drug-resistant bacteria can be easily acclimated during degradation of PCs (Xiong et al., 2018). Microalgae-based biotechnologies have shown potential applications for advanced treatment of different wastewaters such as municipal wastewater, swine wastewater, poultry litter anaerobic digestate, industrial wastewater, and aquaculture wastewater (Yu et al., 2015; Zhuang et al., 2020). Additionally, microalgae can utilize the nutrients and carbon sources of wastewaters for their cellular metabolisms with generating biomass for production of bioenergy and high value-added compounds (Perez-Garcia et al., 2011; Fernandes et al., 2017). Microalgal treatment of PCs needs further exploration to achieve potential application under current scenario; in particular, more attention should be paid on the development in the performance for removal of nutrients and PCs from real wastewaters.

In this study, the microalgal degradation of a frequently found PC, doxylamine from real wastewater, has been investigated. Doxylamine has been frequently detected in different water sources with concentrations of ng L^−1^ to μg L^−1^. Earlier studies mainly investigated the formation dynamics of N-nitrosodimethylamine (NDMA) from doxylamine since NDMA is a highly carcinogenic product and can be easily formed during oxidation of amine-based PCs (Shen and Andrews, 2011). Rare investigations have focused on microalgal removal of doxylamine from real wastewater. Therefore, the current study aims to investigate the removal efficiencies of doxylamine by microalgae from municipal wastewater, the effect of doxylamine on the growth of microalgae, and the effect of doxylamine on the microalgal performance for treatment of COD, TN, and TP.

## Materials and Methods

### Chemicals

Doxylamine succinate salt with a molecular weight 338.46 g mol^−1^ has been obtained from Sigma-Aldrich (St. Louis, USA). Compositions of mobile phase were acetonitrile, water, and formic acid, which are at HPLC-grade and were purchased from Thermo Fisher Scientific and Mallinckrodt Baker Inc. (USA).

### Removal of Doxylamine by *Scenedesmus obliquus* From Real Wastewater

The removal capability of a widely distributed freshwater microalgal species, *S. obliquus*, for doxylamine from real wastewater has been investigated. The microalgal inoculum has been cultivated in Erlenmeyer flasks containing sterilized Bold’s Basal Medium (BBM, 150 ml) under a continuous light source with an intensity of 45–50 μmol photons m^−2^ s^−1^ for 7 days in a shaking incubator (150 rpm). The temperature was 27°C. The microalgal cells were harvested by centrifugation (4,000 rpm), and the biomass was washed three times with distilled water before its further use.

Batch experiments to investigate the removal of doxylamine (1 mg L^−1^) from real wastewater by *S. obliquus* were conducted in 250-ml Erlenmeyer flasks containing 150 ml of raw wastewater inoculated with 1.5% of microalgal suspension with an optical density at 680 nm (OD_680_) of 1.0. The reduction of doxylamine in wastewater was examined by supplying the culture flasks with the same amount of doxylamine without inoculum of microalgae. The effect of sodium bicarbonate (NaHCO_3_, 2 g L^−1^) was also investigated since biocarbonate has been found to be an enhancement strategy not only for microalgal growth but also for promoting the nutritional treatment efficiency (Pancha et al., 2015; Zhai et al., 2020). On the other aspect, high cost of carbon dioxide (CO_2_) capture and transportation and CO_2_ loss for microalgal cultivation have enabled people for searching alternative solutions to CO_2_ fixation (Chi et al., 2011). The bicarbonate-carbon cycling system has been proposed as an advanced approach (Song et al., 2019). All the experiments were conducted in triplicate.

### Determination of Microalgal Growth and Biochemical Characteristics

Growth of *S. obliquus* was determined according to the changes of the microalgal culture at OD_680_ (Xiong et al., 2020). In a brief, the cell numbers of the microalgal cultures with different absorbance were counted using a Countess II Automated Cell Counter (Thermo Fisher Scientific, USA). A linear relationship between microalgal cell numbers and OD_680_ has been found as follows:

$$\begin{array}{l} \mathrm{Cell numbers of}\;S.obliquus\;\left(10^{7}\;{\mathrm{ml}}^{-1}\right)=1.3914\times {\mathrm{OD}}_{680}+\\ 0.0038\;\left(R^{2}=0.999\right) \end{array}$$ (1)

The specific growth rate (μ) was calculated using the following equation:

$$\mu =\frac{\ln N_{2}-\ln N_{0}}{t_{2}-t_{0}}$$ (2)

where *N*_2_ is the dry cell weight at time *t*_2_ and *N*_0_ is the dry cell weight at time *t*_0_ (day 0).

Total chlorophyll and carotenoid content of *S. obliquus* were measured according to an earlier reported protocol (Kurade et al., 2016). Carbohydrate of microalgal biomass was analyzed using phenol-sulfuric acid method with glucose as a standard, and amount of the protein was measured using Bradford assay (Xiong et al., 2019).

### Analytical Measurement of Doxylamine, COD, and Nutrients

Samples for analysis of doxylamine have been taken at regular intervals of 0, 2, 4, 6, 8 and 10 days, which were firstly centrifuged and then filtered using 0.45-μm membrane filters (Pall Life Sciences, USA). Twenty-microliter solution has been injected into a high-performance liquid chromatography (HPLC) equipped with a UV-visible detector (Waters 2695, USA). The running mobile phase consisted of acetonitrile, water, and formic acid at a ratio of 10:90:0.1 (v/v/v), and the flow rate was 0.8 ml min^−1^.

Kinetics analyses of doxylamine removal have been conducted using zero-order model, first-order model, and second-order model based on the following equations:

$$C_{t}=-kt+C_{0}$$ (3)

$$\ln C_{t}=-kt+\ln C_{0}$$ (4)

$$\frac{1}{C_{t}}=kt+\frac{1}{C_{0}}$$ (5)

where *C*_0_ is the initial amount of doxylamine, *C*_t_ is the residual concentration at time *t*, *k* is the removal rate constant (day^−1^), and *t* refers to the sampling time. Equation (3) presents zero-order kinetic reaction; Eq. (4) is for first-order kinetic reaction; and Eq. (5) is for calculation of second-order kinetic reaction parameters.

COD of wastewater was determined with a reference to the guideline of APHA 5220D, which uses potassium hydrogen phthalate for standard curve. TN and TP were measured using the kits named Test N Tube Total Nitrogen Reagent Set (2–150 mg L^−1^) and the phosphorus (total) TNT Reagent Set (0.06–3.5 mg L^−1^). All the kits were purchased from Hach (USA), and the analysis was done according to the manufacturer’s protocol. pH of the samples was determined using an Orion Star A321 pH portable meter (Thermo Fisher Scientific, USA).

## Results and Discussion

### Growth Pattern of *Scenedesmus obliquus*

**Figure 1:**
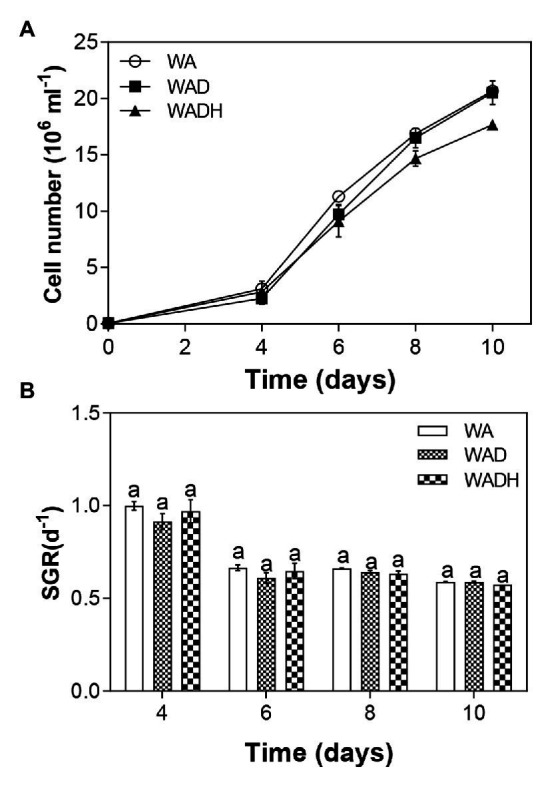
Cell numbers **(A)** and specific growth rate **(B)** of *Scenedesmus obliquus* in wastewater (WA), wastewater with doxylamine (WAD), and wastewater with addition of doxylamine and biocarbonate (WADH) during 10 days of cultivation.

The growth rate of *S. obliquus* with and without doxylamine in wastewater is shown in Figure 1. The density of microalgal cells have been calculated from Eq. (1), and were 20.67 × 10^6^ ml^−1^, 20.51 × 10^6^ ml^−1^, and 17.65 × 10^6^ ml^−1^ for WA (wastewater with inoculum of microalgae), WAD (wastewater with addition of microalgae and doxylamine), and WADH (wastewater with addition of microalgae, doxylamine, and bicarbonate), respectively (Figure 1A). The results indicated that the exposed amount of doxylamine in wastewater negligibly influenced the growth of *S. obliquus*. Specific growth rate (SGR) of *S. obliquus* in different experimental conditions was calculated form Eq. (2), and have been shown in Figure 1B. There was no observable difference of SGR with the effect of either doxylamine or the combination of doxylamine and bicarbonate during 10 days of cultivation. The negligible difference of microalgal growth and SGR influenced by doxylamine indicated that *S. obliquus* is a tolerant species toward the treatment of doxylamine-polluted wastewater. Earlier studies also demonstrated low concentrations of levofloxacin, amoxicillin, and ciprofloxacin (Xiong et al., 2017; Xie et al., 2020). For example, the growth of a green microalga, *Chlorella vulgaris*, was not influenced with exposure up to 5 mg L^−1^ levofloxacin (Xiong et al., 2017). Xie et al. (2020) investigate the effect of ciprofloxacin on a *Chlamydomonas* sp., which showed that there was no significant alteration of microalgal growth under 10 mg L^−1^ ciprofloxacin. Liu et al. (2016) demonstrated that there was a hermetic effect of amoxicillin on the growth of a cyanobacteria, *Microcystis aeruginosa*. All these results indicated the engineering feasibility of microalgae in different conditions. However, there are many studies showing the inhibitory effect of various PCs on microalgal growth. For example, Li et al. (2020) found that the growth of *Chlorella pyrenoidosa* was significantly inhibited by roxithromycin from 0.25 mg L^−1^ to 2 mg L^−1^ (Li et al., 2020). A mixture of sulfamethazine and sulfamethoxazole significantly inhibited the growth of *S. obliquus* at concentrations from 0.05 mg L^−1^ to 0.5 mg L^−1^ (Xiong et al., 2019). Transcriptomic analysis of the microalgal cells exposed to high concentrations of PCs indicated that PCs downregulated the expressions of genes involved in DNA replication and repair process, biosynthesis of biochemicals (steroids, sesquiterpenoid, fatty acids, triterpenoid), and photosynthesis, thus causing toxicity to microalgal cells (Wei et al., 2019; Guo et al., 2020).

### Changes of Microalgal Pigments Influenced by Doxylamine

**Figure 2:**
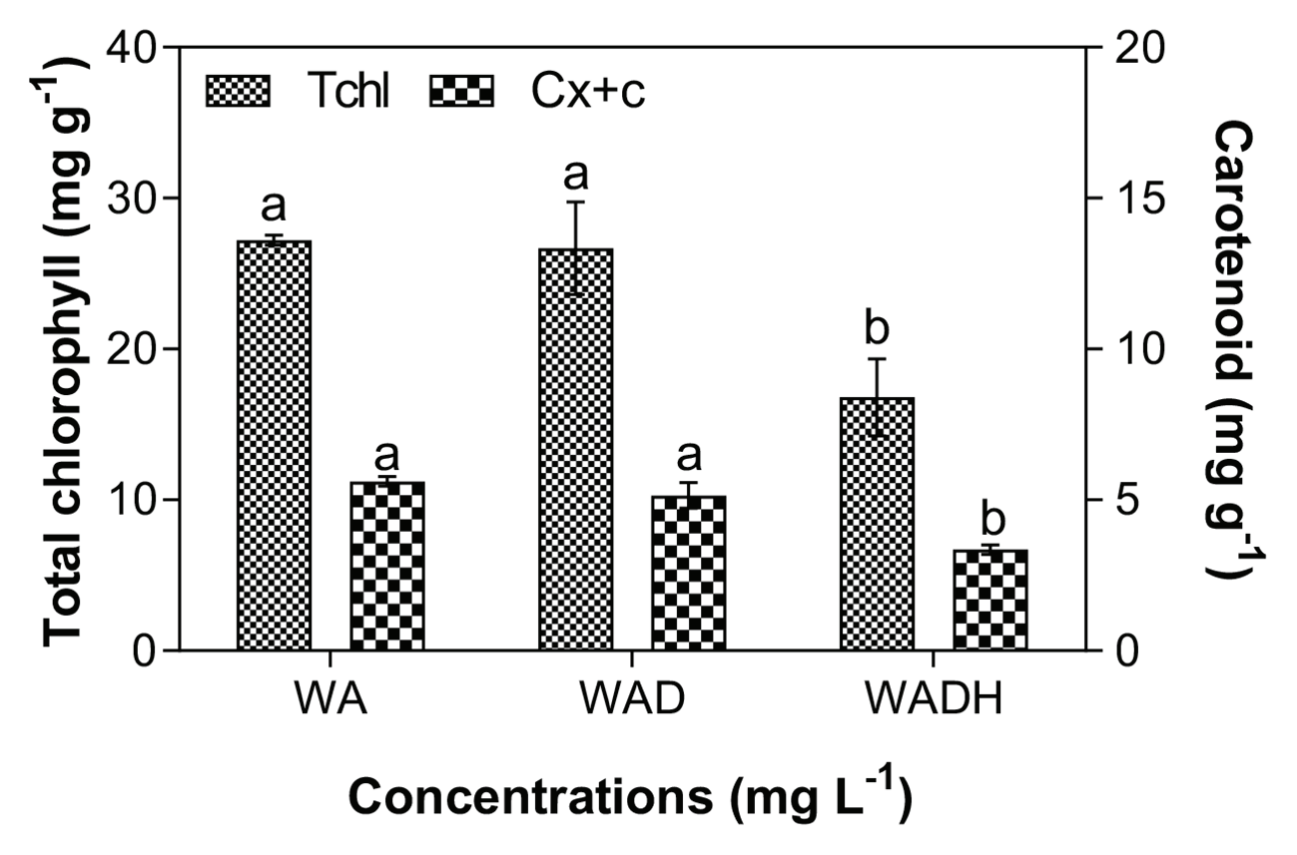
Total chlorophyll and carotenoid content of *Scenedesmus obliquus* after cultivation in wastewater (WA), wastewater with doxylamine (WAD), and wastewater with addition of doxylamine and biocarbonate (WADH).

Photosynthesis of microalgae mediated by chlorophyll and carotenoid plays essential roles to convert light energy, carbon dioxide, and water into microalgal biomass. Thus, evaluation of pigment production helps to understand the overall cellular metabolic activities (Blossom et al., 2019; Stock et al., 2020). As shown in Figure 2, total chlorophyll of *S. obliquus* was 27.21, 26.68, and 16.81 mg g^−1^ in wastewater, doxylamine polluted wastewater, and wastewater with doxylamine and sodium biocarbonate, respectively, whereas the amount of carotenoid showed a similar trend with a final production of 5.62, 5.15, and 3.35 mg g^−1^. The result agreed with the microalgal growth pattern, which indicated that there was no significant effect of doxylamine (1 mg L^−1^) on the microalgal photosynthesis. The decreased content of microalgal chlorophyll is consistent with earlier studies. For example, phytoplankton photosystem II (PSII) efficiency (*Fv*/*Fm*) of a marine microalga, *Nannochloropsis salina*, was slightly inhibited with the addition of 2 g L^−1^ bicarbonate (White et al., 2013). *Fv*/*Fm* of a marine green microalga *Tetraselmis subcordiformis* was significantly inhibited with 5 g L^−1^ bicarbonate (Qi et al., 2019). Sampathkumar and Gothandam (2019) demonstrated that chlorophyll and carotenoid content of a green microalga, *C. vulgaris*, increased at low concentration of bicarbonate (0–150 mM) and decreased at 200 mM bicarbonate (Sampathkumar and Gothandam, 2019). High concentrations of bicarbonate induced adverse effects on photosynthetic pigments, which can be due to energy interactions for photosynthetic carbon dioxide fixation and energy-consuming metabolic pathways since assimilation of bicarbonate involves an active transport (Srinivasan et al., 2018; Qi et al., 2019).

### Removal of Doxylamine by *Scenedesmus obliquus* From Real Wastewater

**Figure 3:**
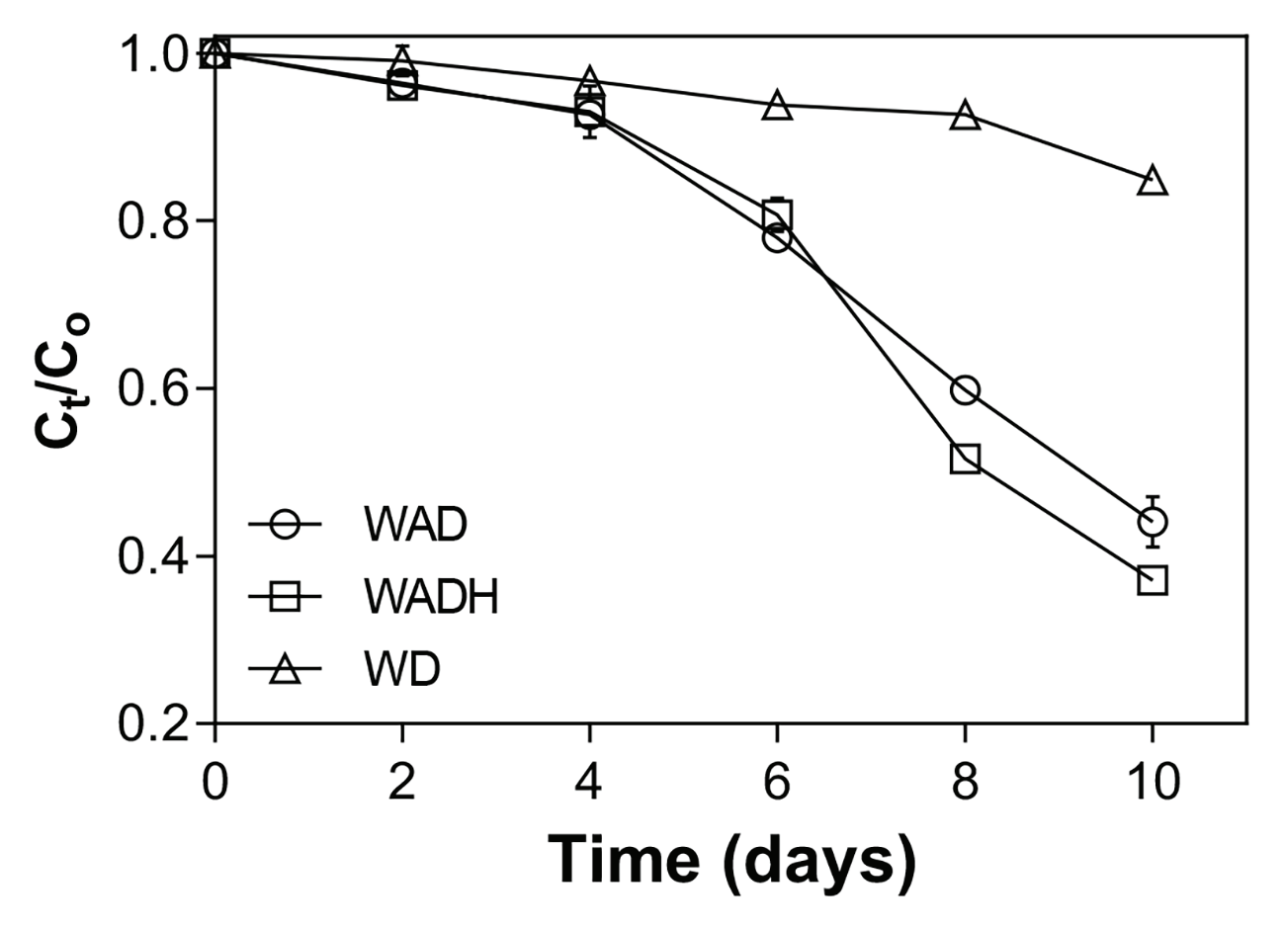
Removal of doxylamine by *Scenedesmus obliquus* in wastewater (WAD), wastewater with addition of bicarbonate (WADH), and wastewater without inoculation of microalgae (WD).

Figure 3 showed the removal of doxylamine from real wastewater by *S. obliquus*. There was 15% decrease of the exposed doxylamine concentrations in the wastewater without inoculum of microalgae, while growth of *S. obliquus* removed 56% doxylamine (1 mg L^−1^) after 10 days of cultivation, and addition of sodium bicarbonate increased the removal up to 63%. Removal kinetics of doxylamine removal at different experimental conditions were analyzed using zero-order, first-order, and second-order reaction models (Table 1). The removal kinetic constant (*k*, day^−1^) of doxylamine from wastewater was 0.057, 0.064 and 0.014 day^−1^ for wastewater with microalgae, wastewater without microalgae and bicarbonate, and wastewater without microalgae, respectively, and half-lives (*T*_1/2_, day) increased from 8.31 days to 36.3 days, which were calculated by zero-order kinetic reaction (*r* ^2^ = 0.87–0.99). First-order kinetic analysis showed the removal kinetic constant (*k*, day^−1^) of doxylamine were 0.12, 0.16, and 0.02 day^−1^ with half-lives (*T*_1/2_, day) of 5.56, 4.33, and 34.15 (*r* ^2^ = 0.86–0.99). Second-order kinetic reaction constant (*k*, day^−1^) was 0.1224, 0.1675, and 0.0166 with half-lives (*T*_1/2_, day) of 10.26, 8.34, and 59.79 (*r* ^2^ = 0.94–0.95) for WAD, WADH, and WD, respectively. Removal of doxylamine using microalgae from real wastewater has been rarely reported. However, numerous studies have demonstrated the removal capacity of microalgae for various PCs. Microalgae-based biotechnologies have been proven as a promising tool toward effective treatment of diverse organic and inorganic pollutants (Villar-Navarro et al., 2018; Sutherland and Ralph, 2019). For example, Vo et al. (2020) found that *Chlorella* sp., can remove 16–58% of tetracycline, sulfamethoxazole, and bisphenol A, and the removal efficiency was enhanced up to 99% with a cometabolic mechanism (Vo et al., 2020). It has demonstrated that the high-rate algal ponds (HRAPs) removed 22–90% of 26 PCs including acetaminophen, ibuprofen, and oxybenzone from urban wastewater (Matamoros et al., 2015). Instead of upflow anaerobic sludge blanket in the wastewater treatment plant, high-rate algae ponds have been used as a tertiary treatment for nutrients and other contaminants from wastewater, which showed 15 and 50% higher removal of diclofenac and some specific antibiotics and diuretics (Villar-Navarro et al., 2018).

**Table 1:** Kinetic parameters of doxylamine (1 mg L^−1^) degradation by *Scenedesmus obliquus* in different experimental conditions.

Experiments	Zero-order kinetic reaction		
	*k* (day^−1^)	*T*_1/2_ (day)	*r* ^2^
WAD	0.057	9.30	0.99
WADH	0.064	8.31	0.97
WD	0.014	36.30	0.87
**Experiments**	**First-order kinetic reaction**		
	***k* (day** ^**−1**^ **)**	***T***_**1/2**_ **(day)**	***r*** ^**2**^
WAD	0.1247	5.56	0.99
WADH	0.1601	4.33	0.97
WD	0.020	34.15	0.86
**Experiments**	**Second-order kinetic reaction**		
	***k* (day** ^**−1**^ **)**	***T***_**1/2**_ **(day)**	***r*** ^**2**^
WAD	0.1224	10.26	0.95
WADH	0.1675	8.34	0.94
WD	0.0166	59.79	0.94

*k*, kinetic removal rate constant (day^−1^); *T*_1/2_, removal half-life (day); *r* ^2^, correlation coefficient; WAD, wastewater with doxylamine and microalgae; WADH, wastewater with doxylamine, microalgae, and biocarbonate; WD, wastewater with doxylamine.

### Removal of COD and Nutrients by *Scenedesmus obliquus*

**Figure 4:**
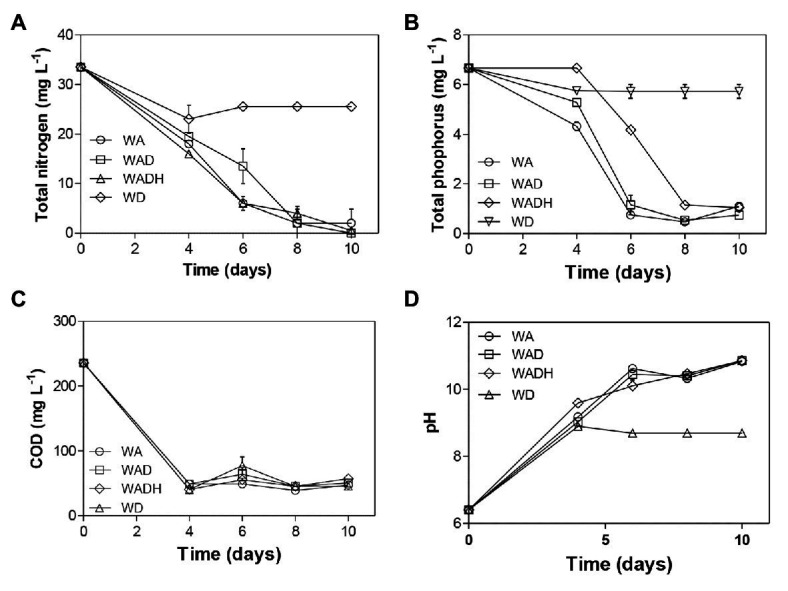
Removal of **(A)** total nitrogen (TN), **(B)** total phosphorus (TP), **(C)** chemical oxygen demand (COD), and **(D)** change of pH in wastewater without microalgae (WD), wastewater with microalgae (WA), wastewater with microalgae and doxylamine (WAD), and wastewater with microalgae, doxylamine, and bicarbonate (WADH).

Figure 4 showed the changes of pH, and removal of COD, TN, and TP in different experimental sets. Investigation of the microalgal capacity for elimination of various contaminants is essential since it can help to screen a robust species. The pH of the raw wastewater (WD) increased from 6.4 to 8.70 after 10 days of cultivation, and pH of wastewaters with cultivation of microalgae (WA), doxylamine (WAD), and/or bicarbonate (WADH) increased up to 10.85 (Figure 4A). Growth of microalgae can increase pH as uptake of inorganic carbon (e.g., HCO_3_^−^) in photosynthesis induces release of hydroxyl ions. Nitrate reduction also causes an increase in pH since denitrification process will consume hydrogen ions. Concentrations of COD decreased from 235.67 mg L^−1^ to 45.67, 47.33, 50.67, and 57.33 mg L^−1^ for WD, WA, WAD, and WADH, respectively. Initial amount of TN was 33.5 mg L^−1^, which achieved 23.9, 94, 100, and 98.5% removal in WD, WA, WAD, and WADH. In case of TP, it was observed that the doses declined from 6.67 mg L^−1^ to 5.72, 1.10, 0.75, and 1.05 mg L^−1^, which equaled to 14.2, 83.5, 88.8, and 84.3% removal, respectively. Microalgae are able to assimilate different nitrogen sources (ammonium, nitrate, and nitrite) from wastewater using different enzymes such as glutamate synthase, glutamine synthetase, glutamate dehydrogenase, nitrite reductase, and nitrate reductase (Perez-Garcia et al., 2011). A microalgal species, *Chlorella* sp., decreased the TN and TP of municipal wastewater from 19.1 to 1.5 mg L^−1^ and from 3 to 0.2 mg L^−1^, respectively, after 9 days of cultivation (Cho et al., 2011; Ji et al., 2013). On the other hand, the presence of other environmental factors such as PCs and heavy metals in wastewaters has significant effect on the removal efficiency of TN and TP. For example, concentrations of 150 μM Cd^2+^, Cu^2+^, or Zn^2+^ inhibited 75% removal of nitrate in *Chlamydomonas mexicana* due to the downregulated activity of glutamine synthetase (Devriese et al., 2001). In the current study, there was no observable effect of doxylamine on the removal efficiency of COD, TN, and TP, indicating the feasibility of *S. obliquus* toward an advanced treatment of wastewater.

### Carbohydrate and Protein Content of *Scenedesmus obliquus* Cultivated in Wastewater

**Figure 5:**
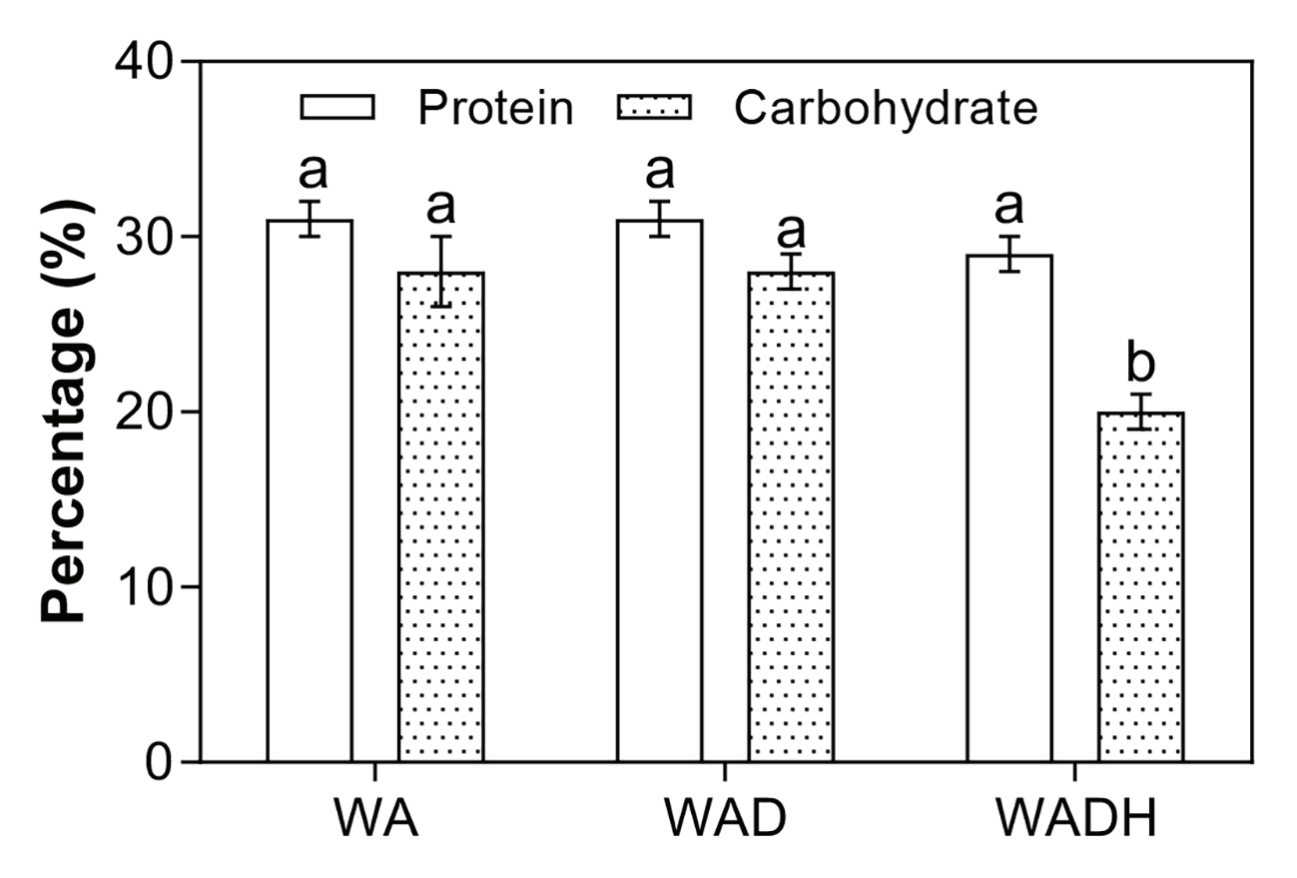
Carbohydrate and protein content of *Scenedesmus obliquus* after cultivation in wastewater (WA), wastewater with doxylamine (WAD), and wastewater with addition of doxylamine and biocarbonate (WADH).

Advantages of microalgae-based wastewater treatment technologies include the high value-added by-products such as carbohydrate and protein-rich microalgal biomass. The effect of doxylamine and bicarbonate on the carbohydrate and protein compositions of the harvested biomass of *S. obliquus* is shown in Figure 5. There were 27, 28, and 26% carbohydrate, and 30, 31, and 33% protein in the microalgal biomass cultivated in WA, WAD, and WADH, respectively, after 10 days of cultivation. Carbohydrate, protein, and lipid are the three main components of microalgal biomass, and changes of their contents can indicate the overall metabolic activities induced by different environments. Earlier studies demonstrated that the PCs such as sulfamethazine and sulfamethoxazole decreased the carbohydrate content of *S. obliquus* since the pollutants disrupted the photosynthesis for carbon fixation and conversion for formation of starch and lignin (Xiong et al., 2019). There was a slight increase in the protein percentage, which is consistent with other studies (Sampathkumar and Gothandam, 2019; Li et al., 2020). Elevated protein content can be caused by the increased synthesis of metabolic and protective enzymes such as degradation and antioxidant enzymes to help in detoxification.

## Conclusion

The treatment capacity of a green microalga, *S. obliquus*, for doxylamine-contaminated wastewater was investigated in this study, and the results showed that *S. obliquus* can grow well under current experimental conditions. There was negligible effect of doxylamine on *S. obliquus* and its biochemical characteristics including chlorophyll, carotenoid, and carbohydrate. In contrast, doxylamine slightly increased the protein content. *S. obliquus* showed high removal capacity toward doxylamine, COD, TN, and TP, indicating its feasibility for the remediation of doxylamine-polluted wastewater. Further studies should be conducted in pilot-scale plants to investigate the engineering application of microalgae-based biotechnology.

## Data Availability Statement

All datasets presented in this study are included in the article/supplementary material.

## Author Contributions

J-QX: conceptualization, funding acquisition, resources, methodology, validation, formal analysis, investigation, visualization, writing – original draft, and writing – review and editing. PC: writing – review and editing. SR: funding acquisition, resources, and writing – review and editing. All authors contributed to the article and approved the submitted version.

### Conflict of Interest

The authors declare that the research was conducted in the absence of any commercial or financial relationships that could be construed as a potential conflict of interest.
